# Demographic, Environmental, and Psychosocial Influences on Resilience Toward Chronic Stress

**DOI:** 10.7759/cureus.67897

**Published:** 2024-08-27

**Authors:** Samantha Johnson, Laura Tunison, Nidhi Thiruppathi, Nicole Humphries, Ibolja Cernak

**Affiliations:** 1 Medical School, Mercer University School of Medicine, Columbus, USA; 2 Integrated Medical Education, Belmont University Thomas F. Frist, Jr. College of Medicine, Nashville, USA

**Keywords:** stress coping, resiliency, stress response, wellness and resilience, environmental risk factors, psychosocial risk factors, demographic risk factors, psychological resilience

## Abstract

As studied previously, chronic stress environments lead to the formation of distinctive resilience groupings when related to individual outcomes among participants. The majority of the population has decreased mental and physical strength during prolonged periods of mental distress but returns to baseline status when those stressors are removed. Others have increased and decreased mental fortitude despite the removal of stressors. Our hypothesis is that certain demographic, environmental, and/or transgenerational aspects are associated with resilience or lack thereof in populations with a history of chronic stress. The end goal is the early identification of at-risk populations to decrease adverse outcomes and improve quality of life. In this review, we looked at 17 studies to gain a greater understanding of which factors influence individual resilience. The factors found to have a positive relationship with resilience were religion, cognitive function, socioeconomic status, marriage, psychological functioning, positive coping mechanisms, and relationships; the negative were medical diagnoses, violence exposure, female sex, stressors/trauma, disaster exposure, and negative coping mechanisms. During our research, we found that transgenerational aspects such as race/ethnicity, occupation, education, age, substance use, and physical location had mixed results across multiple studies. These findings suggest the need for future original research to allow for a definitive understanding of populations resilient to chronic stress.

## Introduction and background

Over the last 40 years, there has been an increased focus in the field of medicine on the disease processes that occur after chronic stress and trauma. In 1980, major depressive disorder, post-traumatic stress disorder, and anxiety were included in the DSM-III for the first time as official diagnoses [1]. This validation paved the way for research initiatives aimed at determining factors that increase the likelihood of progressing to a chronic psychological disease state versus recovering from life stressors. Adding to the complexity of this investigation, researchers identified multiple responses to chronic stress. Some responses included those who experience (a) maladaptive responses to stress but have a return to baseline after the stressor is removed, (b) continual distress after the stressor is removed, and (c) positive mental responses both during and after a stressor is introduced [2]. Despite this insight, a definitive list of factors pinpointing the exact causes of an individual’s increased or decreased resilience to stress remains elusive.

In this literature review, Hellewell and Cernak’s research on measuring innate resilience [2] serves as the basis for understanding the dynamic nature of stress and individual responses to stressors. Using stress markers paired with cognitive function tests to assess responses during and after stressful situations, they identified distinctive stress profiles. These included individuals who respond with decreased mental fortitude in response to stress long-term, those who have an easily reversible negative response to stress, and those who have improved mental function and fortitude after the onset of stress. It was noted that a majority of the population, 68%, were able to bounce back and have an easily reversible reaction to stress [2]. Building on this, we hypothesized that certain demographic, environmental, and transgenerational factors are associated with resilience in individuals undergoing chronic stress. We defined chronic stress as prolonged periods of mental and emotional distress occurring over the course of months to years.

The goal of this review is to determine which factors are associated with populations immune to chronic stress and its deleterious outcomes. With these factors defined, the development of early intervention protocols focused on high-risk patient populations can be established. These interventions will allow for more intensive monitoring of patients with maladaptive responses who are at risk of developing long-term physical and mental dysfunction after experiencing chronic stress. It will also allow appropriate counseling to be offered to educate patients on which factors could be modified to benefit their long-term health and quality of life.

This article was previously presented as a poster presentation at the 2022 Summer Research Enrichment Symposium on August 8, 2022.

## Review

Data collection and analysis

Search Strategy

We performed keyword searches (Table 1) of original research articles and literature reviews in PubMed via EndNote Basic software with the aim of answering the following Population, Intervention, Comparison, Outcomes (PICO) question: “Are certain demographic, environmental, and/or transgenerational aspects associated with increased resilience in patients with a history of chronic stress?” The search terms for our queries were derived directly from these predefined PICO variables. The population was defined as patients with a history of chronic stress. The intervention included demographic, environmental, and/or transgenerational aspects. There was no comparison based on the open-ended nature of the research question. Last, the overall outcome was increased resilience.

**Table 1:** Keyword search ^*^: number of articles resulting from PubMed search via EndNote Basic software after exclusion of duplicates

Search query	Search yield^*^
Childhood AND demographic AND chronic stress AND resilience	18
Cognition AND demographic AND chronic stress AND resilience	9
Demographic AND environmental influences on resilience	69
Environmental AND resilience AND chronic stress	271
Neurocognition AND demographic AND chronic stress	4
Neurocognition AND demographic AND resilience	7
​​Psychological Resilience AND Neurocognitive Performance	14
Transgeneration AND chronic stress AND demographic	6
Transgeneration AND resilience	80
Transgenerational AND resilience AND chronic stress	3
Trauma AND demographic AND generational	22

Study Selection

**Figure 1:**
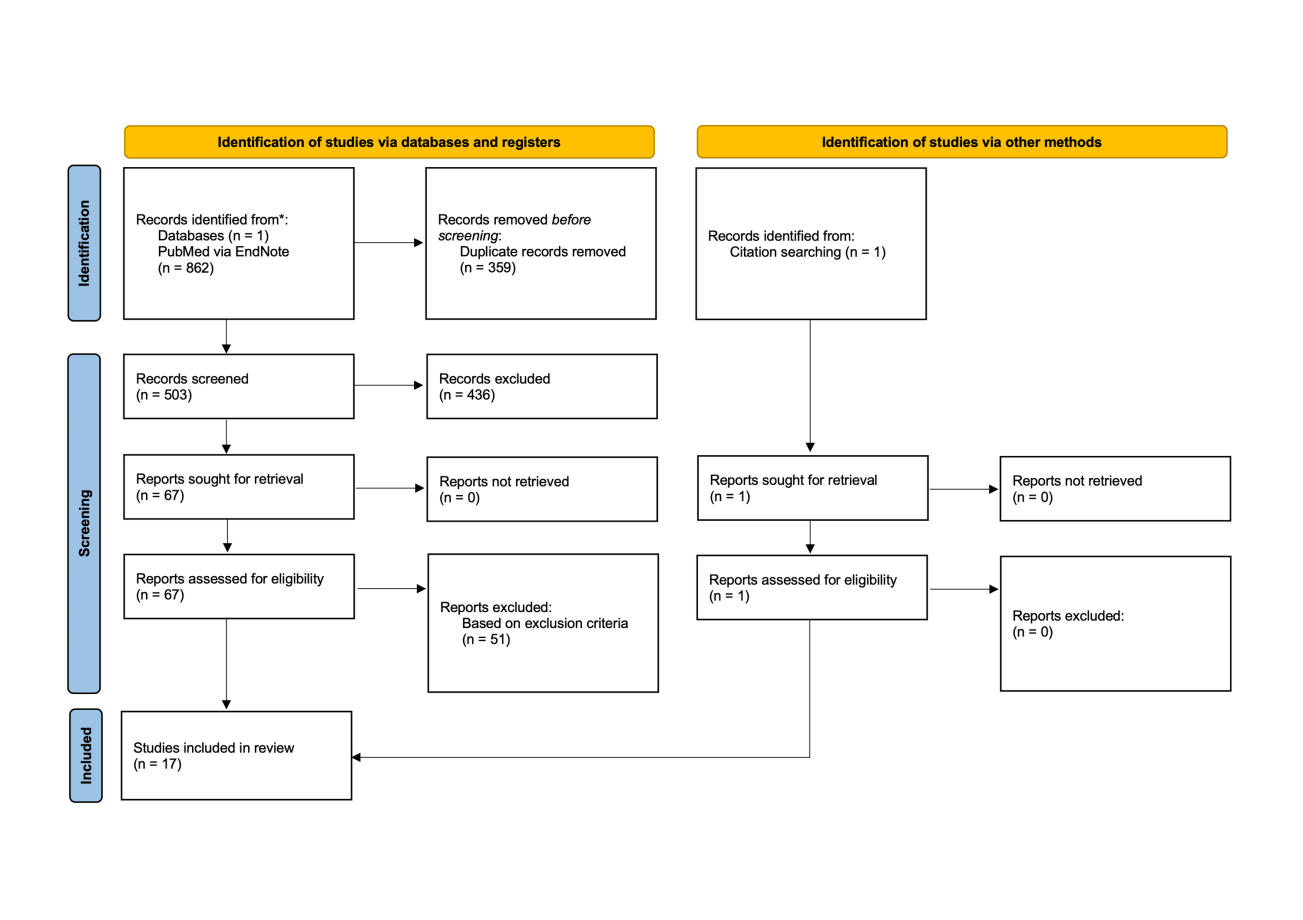
PRISMA 2020 flow diagram for reviews PRISMA: Preferred Reporting Items for Systematic Reviews and Meta-Analysis

To summarize the selection methods used during this literature review, a flow diagram was created using the Preferred Reporting Items for Systematic Reviews and Meta-Analyses (PRISMA) 2020 guidelines to summarize the selection methods used during this literature review (Figure 1).

The initial search included studies from 1990 to 2022 and yielded 862 results, from which all automatically detected duplicate records were removed. The abstracts of the remaining 503 articles then underwent a primary review for relevance to the proposed research question. During this time, one article was identified and assessed based on citation searching. A set of inclusion and exclusion criteria (Table 2) were developed to allow for data relevant to the hypothesis to be assessed. These criteria as established by the proposed PICO question were included to better understand which individual factors found in humans influence resilience when compared between diverse groups of people. Studies that were unable to note any limiting factors to their research were deemed unreliable resources. Research with limited sample sizes was unable to be generalized, therefore decreasing their external validity. Last, many studies had keywords matching those noted in Table 1 but were not relevant to the hypothesis (e.g., those heavily focused on genetic influences). These studies were discarded and the remaining 68 articles were assessed in a secondary, more detailed review. This review evaluated for quality and bias by using the Critical Appraisal Skills Programme checklist for systematic reviews. This was paired with the inclusion and exclusion criteria outlined in Table 2 and resulted in 17 articles remaining as the primary focus of this study.

**Table 2:** Selection criteria used for secondary review

Inclusion criteria	Exclusion criteria
Limiting factors in study	Lack of limiting factors in study
Sufficient sample size; >15 participants	Limited sample size; <15 participants
Pertinent to current research question	Lack of pertinence to research question
Individual-based	Population-based
Between-group comparisons	Within-group comparison
Human study	Zoologic/ecologic study
Demographic, environmental, transgenerational influences	Genetic influences

The data of the remaining articles (Table 3) was categorized into groups and subgroups and then assigned to one of three overarching classifications: demographic, environmental, or psychosocial (with psychosocial replacing transgenerational due to an absence of conclusive transgenerational research studies). By combining multiple studies and standardizing their categorization, we were able to gain a general understanding of the factors consistently found to affect resilience despite the diversity of the research studies.

**Table 3:** Studies remaining after secondary review PTSD: post-traumatic stress disorder

Author(s)	Year	Title	Journal	Objective	Methodology	Key findings
Abbott et al. [3]	2022	Latent profile analysis of stress and resilience among rural women: a cross-sectional study	Public Health Nurs	To identify what distinct characteristics relate to resilience in rural women undergoing stress	Cross-sectional study	Rural women experiencing higher levels of stress are less likely to afford necessities and are more likely to be employed while those with lower stress are more likely to have resilient characteristics
Bethell et al. [4]	2014	Adverse childhood experiences: assessing the impact on health and school engagement and the mitigating role of resilience	Health Aff	To identify the prevalence and future ramifications of adverse childhood experiences	Cross-sectional study	Children who experience adverse events are more likely to have long-term health complications and are less likely to excel academically
Bonanno et al. [5]	2007	What predicts psychological resilience after disaster? The role of demographics, resources, and life stress	J Consult Clin Psychol	To identify which factors contribute to psychological resilience following a disaster	Cross-sectional study	Resilience can be predicted based on demographic, socioeconomic, psychological, and health factors among post-disaster participants
Cheng et al. [6]	2019	Trajectories of PTSD symptoms among children who survived the Lushan earthquake: a four-year longitudinal study	J Affect Disord	To explore PTSD symptoms and trajectories in children after a natural disaster	Longitudinal study	Social support has a positive overall impact on PTSD symptoms in post-disaster children
Cherry et al. [7]	2018	Spirituality, humor, and resilience after natural and technological disasters	J Nurs Scholarsh	To identify how spirituality and humor influence resilience in populations exposed to disasters	Cross-sectional study	Spiritual support and humor have a positive association with resilience while lack of charitable work and lower income negatively impact resilience
Durbin et al. [8]	2019	Are resilience and perceived stress related to social support and housing stability among homeless adults with mental illness?	Health Soc Care Community	To examine how stress and resilience are perceived by homeless, mentally ill adults after obtaining improved social support	Longitudinal study	Social support and social functioning are associated with increased resilience and decreased stress
Feder et al. [9]	2016	Risk, coping and PTSD symptom trajectories in World Trade Center responders	J Psychiatr Res	To study post-disaster symptoms while identifying risk factors and protective characteristics associated with resilience	Longitudinal study	Coping mechanisms, social support, disaster exposure, and health concerns are associated with varying PTSD trajectories in post-disaster participants
Kellerman [10]	2001	Psychopathology in children of holocaust survivors: a review of the research literature	Isr J Psychiatry Relat Sci	To evaluate psychopathology in the offspring of Holocaust survivors	Literature review and analysis	There is inconclusive evidence as to whether children of Holocaust survivors have increased psychopathology
Lucier-Greer et al. [11]	2014	Adolescent mental health and academic functioning: empirical support for contrasting models of risk and vulnerability	Mil Med	To examine the relationship between different risk factors and adolescent well-being in children of military families	Cross-sectional study	Risk factors and social stressors have a negative impact on adolescent well-being by increasing adverse outcomes
Macedo et al. [12]	2018	Parental bereavement in young children living in South Africa and Malawi: understanding mental health resilience	J Acquir Immune Defic Syndr	To explore predictors of resilience in children whose parents are deceased	Longitudinal study	Social support and lack of exposure to violence are associated with increased resilience in parentally bereaved children
Ntatamala and Adams [13]	2022	The correlates of post-traumatic stress disorder in ambulance personnel and barriers faced in accessing care for work-related stress	Int J Environ Res Public Health	To determine factors that decrease resilience and increase PTSD	Cross-sectional study	Stressors, health conditions, substance use, living in an urban environment, and working as ambulance personnel are associated with increased PTSD prevalence
Orcutt et al. [14]	2014	Prospective trajectories of post-traumatic stress in college women following a campus mass shooting	J Trauma Stress	To evaluate the trajectory of PTSD and psychological functioning after experiencing mass violence	Longitudinal study	Increased emotional regulation and functioning prior to mass violence is associated with higher resiliency
Osofsky et al. [15]	2015	Trajectories of post-traumatic stress disorder symptoms among youth exposed to both natural and technological disasters	J Child Psychol Psychiatry	To explore PTSD symptoms and trajectories in adolescents after natural and technological disasters	Longitudinal study	Female sex, older age, and exposure to natural and technological disasters are associated with decreased resilience among adolescents
Raman et al. [16]	2021	Community violence, PTSD, hopelessness, substance use, and perpetuation of violence in an urban environment	Community Ment Health J	To identify the role that violence and substance use plays on resilience	Cross-sectional study	Female sex, substance use, and hopelessness are associated with decreased resilience and increased PTSD prevalence
Santarelli et al. [17]	2017	An adverse early life environment can enhance stress resilience in adulthood	Psychoneuroendocrinology	To determine whether psychosocial stress experienced in childhood has an association with resilience in adulthood	Laboratory study, analysis of mice resilience	Early life adversity is associated with increased resilience to adversity in adulthood
Toledano-Toledano et al. [18]	2021	Psychosocial factors predicting resilience in family caregivers of children with cancer: a cross-sectional study	Int J Environ Res Public Health	To identify which sociodemographic and psychosocial factors are associated with resilience	Cross-sectional study	Social support and positive adaptation processes are associated with increased resilience in caregivers of children with cancer
Wingo et al. [19]	2010	Psychological resilience and neurocognitive performance in a traumatized community sample	Depress Anxiety	To identify factors promoting resilience in traumatized participants	Cross-sectional study	Enhanced non-verbal memory is associated with increased resilience in abused and traumatized participants

Results

During our review, we found that multiple studies provided specific factors associated with resilience (Table 4). However, our focus on demographic, environmental, and psychosocial factors prevented definitive causation from being proved based on the Bradford Hill Criteria [20]. The criteria require specific characteristics such as consistency between studies, ruling out confounding factors, and specificity in experimental design to be met prior to noting a causal relationship between factors [20]. Given the nature of this research, observational, cross-sectional studies were the sole sources of information. This resulted in our establishment of associations between factors without proving causation. Despite this, many important influences were found to facilitate positive and negative outcomes regarding overall resilience to chronic stress.

**Table 4:** Factors affecting resilience

	Resilient	Non-resilient	Inconclusive
Demographics	Marriage, religion, socioeconomic status	Female sex, Hispanic ethnicity, medical diagnoses	Age, education, racial minority, stressful occupation
Environmental	None	Disaster exposure, stressors/trauma, violence exposure	Geographical location, substance use
Psychosocial	Positive psychological functioning, social support	Negative coping mechanisms, negative psychological functioning	None

Demographic Factors

Our review of the literature suggests that multiple demographic factors may play a role in increasing or decreasing a person's resilience toward chronic stress. However, the available research on these factors is often contradictory, with some studies suggesting that a given factor may enhance resilience, while others suggest that the same factor may inhibit resilience. Considering this inconsistency, our review focused on identifying the demographic factors that were consistently associated with resilience as well as those for which the available evidence was inconclusive.

We found that the demographic factors most commonly associated with resilience throughout our review include marital status, religion, cognitive function, medical diagnoses, sex, and socioeconomic status. Two studies indicate that being married positively affects resilience, with Abbott et al. reporting that married individuals among rural women in the southeastern United States “were about half as likely to be in the Moderate Stress profile compared to the Low Stress profile” [3] and Toledano-Toledano et al. finding married family caregivers of children with cancer to be more resilient than those sharing a domestic partnership [18]. The latter study also showed greater resilience in Catholic family caregivers, while Cherry et al. attributed this positive correlation in the context of post-disaster resilience to spiritual support regardless of organized religious affiliation [7]. Particular cognitive functions, being a quick learner and having better nonverbal memory, were also found to positively affect resilience in two studies exploring predictors of resilience in traumatized populations [12,19]. Demographic factors found to negatively affect resilience are represented in a larger percentage of the reviewed literature. Seven studies [5,6,8,9,13,18,19] attributed medical diagnoses, including psychiatric, to decreased resilience and six [5,6,9,15,16,19] to the female sex. Increased socioeconomic status in the form of employment [7,12,19], increased income [7,18], and lack of homelessness [8] were all tied to greater resilience throughout five studies. Abbott et al. also overwhelmingly found improved socioeconomic status to be correlated with positive overall outcomes but did state that employment negatively affected resilience during an analysis of rural women [3]. This same study also found that an inability to afford necessities carried a negative impact. As these findings seem to contradict each other, we can say that overall, increased socioeconomic status is positively correlated to resilience.

Due to inconsistent findings across studies, the association between resilience and demographic factors such as age, education, occupation, race, and ethnicity is less clear. There were conflicting results regarding how resilience is related to increasing age. Feder et al. reported that increased age contributed to the chronicity of post-traumatic stress disorder symptoms in adults after a terrorist attack [9] with Osofsky et al. noting similar findings in adolescents after natural disasters [15]. Meanwhile, two studies found that older age had a positive impact on resilience following a terrorist attack [5] and a mass shooting [14]. Regarding education, studies showed that school engagement [4], the number of years spent studying (up to 18 years total) [18], and having a college education [3] had a positive correlation with resilience. However, Bonanno et al. reported “participants with a college education were only about half as likely to be resilient as were participants with less than a high school education” [5]. Additionally, some studies found that government and public service occupations were found to have a negative impact on resilience in both workers [13] and children of these workers [11] while others found a positive correlation [9]. When discussing race and ethnicity, there is no clear-cut answer to whether minority groups are more or less resilient than their counterparts. This was shown when adults who were identified with African American [19] and Asian [5] races were determined to have higher resiliency scores, but adolescents of minority races and ethnicities were found to be less resilient [11]. Meanwhile, non-white and Hispanic ethnicity were consistently shown to have a negative effect on resilience [3,9,14]. These conflicting studies show that many demographic factors are not easily applied as parameters for determining resilience in a specific population. Further research is needed to delve into the complex relationship between these demographic factors and resilience toward chronic stress.

Environmental Factors

Based on consistent research findings across multiple studies, the following environmental factors were determined to decrease resilience to chronic stressors: disaster exposure, stressors/trauma, and violence. Alternatively, physical location and substance abuse had both a positive and negative effect on resilience depending on the research study performed. In terms of disasters, both technological [15] and natural disasters [6,15,21] including earthquakes, famine, oil, spills, and hurricanes had devastating effects on personal resilience. However, other than the location of disasters, the geographical region did not determine population-wide resilience. Living in both rural [3] and urban [13,16] environments was tied to susceptibility toward stress and post-traumatic stress disorder, respectively. Overwhelmingly, stressors and trauma were found to adversely influence future resilience [5,9,12-14,19] with the caveat of matched life stressors [17] having a mitigating effect. This was found in a study performed by Santarelli et al. that reported experiencing early life stressors had an advantageous effect on mice who later experienced adverse adulthood stressors [17]. While this may be an area for future research, there seems to be a lack of generalizability to human populations based on current research data. As violence is a known stress factor, the findings of studies performed by Raman et al. and Orcutt et al. coincided with the notion that stress produces unfavorable outcomes in overall resilience to chronic stress [14,16]. Interestingly, substance abuse was found to have contradictory effects on resilience in studied populations. While environments where substance abuse is prevalent would seem to be categorized as stressors, there were both positive [8] and negative [16] findings regarding how these patient populations respond to stress.

Psychosocial Factors

The study of resilience overwhelmingly reflects on individual personality factors that increase or decrease resilience in a population. The most strongly studied personality factors assessed were not only found through examination of original research but also by examination of methods employed to determine resilience. For example, many articles used surveys as tools for comparing hypothesized resilience factors to a measurable scale to gain a greater association factor between the two. Overall, these scales focus on a person’s internal factors to determine associated risk. The Connor-Davidson Resilience Scale 25 (CD-RISC-25) [22] is one scale in particular that measures self-reported positive psychological factors on a scale from zero to four with 100 having the highest resiliency likelihood based on statistical factor analysis. Based on multiple follow-up studies, these factors of “persistency/tenacity” and “self-efficacy” [22] have remained reliable markers for the ability to cope with stress. Our literature review echoed this sentiment as coping mechanisms, psychological functioning, and relationships were all found to be related to resilience. Four studies indicated that positive psychological functioning in the form of adaptability, emotional clarity, emotional regulation, sense of purpose, optimism, quality of life, and self-efficacy have positive long-term effects [3,9,14,18]. Additionally, the reverse was true in a study by Raman et al. that found hopelessness, a negative psychological factor, to be detrimental [16]. Also, four studies [7,9,13,14] found maladaptive coping mechanisms [13] such as self-blame [9] and avoidance [14] to be unfavorable toward resilience while constructive coping strategies relating to humor [7,9], acceptance [9], and positive reframing [9] were beneficial. Unsurprisingly, social support contributed substantially to resilience throughout the nine studies [3-6,8,9,11,12,18] evaluating the connection between psychosocial factors and resilience to chronic stress.

Transgenerational Factors

Of the literature reviewed, we found that the field of transgenerational and epigenetic research has moved from a theoretical approach to a quantifiable science in recent years with the findings of specific DNA methylation, histone modification, and microRNA expression inherited vertically through animal models [23,24]. Despite these findings, there is still much to learn about transgenerational inheritance in terms of human transmission of genetic factors causing increased or decreased resiliency in subsequent generations.

The field of transgenerational transmission of stressors in humans started after the Holocaust with Sigal and Rakoff’s [25] study of second-generation effects on concentration camp survivors’ children. They found that the secondary effects related more to the “emotionally depleted state of the parents” [25] as opposed to innate differences between children. While this study did not prove that parental stressors limit resiliency in future generations, it did open the door to continued research efforts with the goal of understanding protective and detrimental risk factors. Despite these efforts, there has been no consensus [10,26] on which factors induce a transgenerational response and how this response can be measured in human studies. We attributed this to the limited timeframe of transgenerational research as a whole. Due to initial studies beginning in the 1970s, there has not been enough time to adequately study further than the second generation and their described outcomes. This leaves researchers unable to prove that parental stressors are responsible for generational gene activation and inactivation related to resilience.

Alternatively, transgenerational animal studies have provided substantial results, with many finding sufficient evidence of causation concerning environmental factors and resilience. Most notably, maternal care, early adverse experiences, and parental (specifically paternal) experiences have been found to be the three strongest factors associated with resilience among subsequent generations [24]. Despite having helped bridge the gap between the lack of consistency regarding human transgenerational studies, these studies were excluded from our review as they were not generalizable to the patient population indicated in the inclusion criteria noted previously.

Our review found no definitive evidence of causation associated with transgenerational stressor transmission nor definitive factors tying transgenerational stressors to gene expression in human subjects. Major limitations of human transgenerational studies are based on the lack of longitudinal cohort data over extended periods of time. Without having the necessary time required to procure data sets on third generations of participants and beyond, researchers have been unable to prove that parental stressors are responsible for generational gene activation and inactivation related to resilience. It is our hope that future research will give greater insight into how past stressors can affect future generations and their ability to overcome stress. We propose the need for family-based cohort studies that incorporate multidisciplinary data to provide a holistic understanding of whether certain events or triggers can collectively change the course of future generations.

Discussion

Our review identified conflicting results regarding many demographic and environmental factors that may contribute to resilience, and conclusive data was extremely limited regarding transgenerational factors affecting resilience. Additionally, contradicting evidence regarding the merits of epigenetic resilience factors passed generationally in human studies caused the exclusion of this factor from our final review. Despite this, certain factors were agreed upon by all research articles evaluated such as marital status, medical diagnoses, cognitive function, religion, sex, exposure to disasters, stressors/trauma, and violence. Also, despite not being included in the original research question, psychosocial factors were included as they explain many personal factors that have been associated with contributing to an individual’s resilience.

We believe that this review can help guide future interventions to reduce environmental and psychosocial actions that may inhibit resilience. However, as demographics are generally unchangeable, knowledge of these specific factors will be most important in the context of patient screening. By developing standardized screening tools, patients will be assessed based on risk factors to guide future interventions. This can allow for improvement in monitoring as well as patient-specific care in populations at the highest risk of decreased resilience in response to chronic stress. Taking the inverse of non-resilience or “at-risk” populations can lead us to the assumption of factors that boost or at least contribute to resilience. Our goal is for healthcare professionals to understand what factors can be emphasized or counseled on to decrease the risk of physical and mental illness in their patient populations. By screening new and existing patients for underlying demographic, environmental, and psychosocial factors with standardized intake questionnaires at-risk populations can be identified. Furthermore, care can be given to ensure that these risk factors are not compounded with negative coping mechanisms and, consequently, achieve better overall health outcomes.

This review takes many liberties with the generalization of risk factors and preventative factors for resilience to chronic stress. This is due in part to the lack of research with sufficient evidence of a causal relationship as well as the limited number of original research articles related to our research question. Nearly half of the studies were cross-sectional in nature, which eliminates the ability to prove causation. Furthermore, most of the factors researched do not have the ability to undergo experimentation through randomized controlled, double-blind studies. A prime example of this would be attempting to test whether homelessness causes decreased resilience; it would be not only unethical, improbable, and unreasonable to study this factor in this way but also unlikely that the participant sample size would be appropriate for analysis. These generalizations can be amended with future research that provides a more comprehensive perspective on specific factors associated with increasing or decreasing resilience to chronic stress.

The lack of consistency regarding many of the demographic, environmental, and transgenerational factors evaluated for affecting resilience also emphasizes the need for future research. With more defined longitudinal designs, previously confirmed factors related to resilience can be used by medical professionals to create and implement interventions. Patients noted to have decreased resilience based on standardized screening methods will undergo risk stratification to determine the need for the implementation of risk aversion practices. This will focus on the more at-risk populations, with reliable research backing the focus on screening specific groups. Furthermore, a focus on the distinguished psychosocial aspects will allow the interventions to be customized to increase resilience to chronic stress at the individual level by working to implement coping mechanisms that improve resilience to chronic stress.

## Conclusions

As studied previously, chronic stress environments lead to the formation of three distinctive groupings when related to individual outcomes among participants. The majority of the population has decreased mental and physical strength during times of chronic stress but returns to baseline when stressors are removed. Individuals have positive and maladaptive reactions and have increased and decreased fortitude, respectively, despite the removal of stressors. Our hypothesis, in an attempt to define the factors allowing for these outcomes, was that certain demographic, environmental, and/or transgenerational aspects are associated with resilience or lack thereof in populations with a history of chronic stress. This review analyzed 17 studies to determine which factors were associated with each category as well as their role in resilience. The factors found to have a positive relationship with resilience are religion, cognitive function, marriage, psychological functioning, positive coping mechanisms, socioeconomic status, and relationships; those found to have a negative relationship are medical diagnoses, violence exposure, female sex, stressors/trauma, disaster exposure, and negative coping mechanisms. This review also found that transgenerational aspects such as race/ethnicity, occupation, education, age, substance use, and physical location have mixed results as resilience factors across multiple studies. These findings suggest the need for future original research to allow for a definitive understanding of populations resilient to chronic stress. This research can include longitudinal studies with a focus on transgenerational factors, cross-sectional research designs exploring the relationship between demographic and psychosocial resilience factors, and more intensive research designs examining environmental risk factors between populations.
